# Baseline Functioning and Stress Reactivity in Maltreating Parents and At-Risk Adults: Review and Meta-Analyses of Autonomic Nervous System Studies

**DOI:** 10.1177/1077559516659937

**Published:** 2016-07-26

**Authors:** Sophie Reijman, Marian J. Bakermans-Kranenburg, Regina Hiraoka, Julie L. Crouch, Joel S. Milner, Lenneke R. A. Alink, Marinus H. van IJzendoorn

**Affiliations:** 1 Centre for Child and Family Studies, Leiden University, Leiden, the Netherlands; 2 Leiden Institute for Brain and Cognition, Leiden, the Netherlands; 3 Center for the Study of Family Violence and Sexual Assault, Northern Illinois University, DeKalb, IL, USA

**Keywords:** child maltreatment, review, autonomic nervous system, stress, meta-analysis

## Abstract

We reviewed and meta-analyzed 10 studies (*N* = 492) that examined the association between (risk for) child maltreatment perpetration and basal autonomic activity, and 10 studies (*N* = 471) that examined the association between (risk for) child maltreatment and autonomic stress reactivity. We hypothesized that maltreating parents/at-risk adults would show higher basal levels of heart rate (HR) and skin conductance (SC) and lower levels of HR variability (HRV) and would show greater HR and SC stress reactivity, but blunted HRV reactivity. A narrative review showed that evidence from significance testing within and across studies was mixed. The first set of meta-analyses revealed that (risk for) child maltreatment was associated with higher HR baseline activity (*g* = 0.24), a possible indication of allostatic load. The second set of meta-analyses yielded no differences in autonomic stress reactivity between maltreating/at-risk participants and nonmaltreating/low-risk comparison groups. Cumulative meta-analyses showed that positive effects for sympathetic stress reactivity as a risk factor for child maltreatment were found in a few early studies, whereas each subsequently aggregated study reduced the combined effect size to a null effect, an indication of the *winner’s curse*. Most studies were underpowered. Future directions for research are suggested.

The possibility that dysregulated psychophysiology may serve as a risk factor for child maltreatment has been the topic of long-standing (albeit intermittent) research interest, particularly with respect to the activity of the autonomic nervous system (ANS). The ANS may be relevant in the etiology of child maltreatment because of its role in emotion and behavioral responsiveness (Stemmler, 2004; Sturge-Apple, Skibo, Rogosch, Ignjatovic, & Heinzelman, 2011). Prior research has suggested increased ANS (re)activity in maltreating parents and individuals at risk for perpetrating child maltreatment; however, inconsistent findings across as well as within studies have been noted (McCanne & Hagstrom, 1996). Furthermore, the last review of the literature on this topic was conducted approximately 20 years ago (McCanne & Hagstrom, 1996), and effect size estimates for the association between physiological (re)activity and perpetration and risk for perpetration of child maltreatment have not been assessed using meta-analytic methods. To address this gap in the literature, we reviewed the literature and conducted two meta-analyses: one examining the association between (risk for) child maltreatment and baseline ANS activity levels and another examining the association between (risk for) child maltreatment and ANS stress reactivity.

The ANS is a component of the biological stress system (Stratakis & Chrousos, 1995). It regulates the visceral organs and consists of the parasympathetic and the sympathetic branches whose functions, generally speaking, lead to opposite effects. The parasympathetic division (parasympathetic nervous system (PNS)) slows down heart rate (HR) and stimulates digestion, promoting the conservation and recuperation of energy (i.e., anabolic processes). The sympathetic nervous system (SNS) increases HR and inhibits digestion, mobilizing the body in response to, or in anticipation of, environmental challenges (i.e., catabolic processes; Viamontes & Nemeroff, 2009). The ANS is a well-recognized component of emotion (see Kreibig, 2010, for a review). For instance, in the context of stress, the typical autonomic reaction comprises an increase in HR (determining the rate at which organ tissues receive nutrients such as oxygen from the blood); an increase in sympathetic activity, as reflected by SNS measures such as SC (i.e., sweat gland activity); and a decrease in parasympathetic activity, as reflected by a decrease in HR variability (HRV; a PNS index). These physiological reactions facilitate behavioral responses to (anticipated) demands from the environment.

The acoustic characteristics of infant cry sounds tend to elicit stressful feelings in parents, accompanied by increases in HR and sympathetic activity (e.g., Frodi, Lamb, Leavitt, & Donovan, 1978; Joosen et al., 2013a). While this may be how infant crying prompts regulatory caregiving behavior, thereby promoting the infant’s survival, the paradoxical fact remains that it is also associated with an increased risk for child abuse, even infanticide (Barr, 1990; Soltis, 2004). Autonomic responses to stressful stimuli (e.g., infant cry sounds) that deviate from normative responses reflect abnormal (e.g., overly strong) reactions to stressors and as such may contribute to inappropriate behavioral responses (e.g., use of excessive force). Several studies support this notion. For instance, mothers’ SC hyperreactivity to infant cry sounds predicted harsh parenting responses 9 months later (Joosen, Mesman, Bakermans-Kranenburg, & Van IJzendoorn, 2013b). In addition, less sensitive mothers showed weaker HRV decreases in response to infant cry sounds than highly sensitive mothers (Joosen et al., 2013a).

Stress theory has focused not only on momentary ANS reactivity to stressors but also on chronic autonomic activation due to the anticipation of, delayed recovery from, or repeated recall of stressors (Brosschot, Pieper, & Thayer, 2005; Juster, McEwen, & Lupien, 2010). Rather than providing real-time insight into parents’ immediate responses to stress (as in the case of ANS reactivity), sustained autonomic activation may more generally reflect a person’s capacity for emotion regulation. Although research in this area is scarce, converging findings exist. For instance, quick cardiovascular recovery from negative emotions has been found in highly resilient individuals (Tugade & Fredrickson, 2004). Moreover, high resting HRV has been linked with adaptive emotion regulation (see Appelhans & Luecken, 2006, for a review). Consistently, highly sensitive mothers showed lower resting HR and higher resting HRV compared to less sensitive mothers (Joosen et al., 2013a). Harsh mothers’ SC did not recover after listening to stressful infant cry sounds, while the SC of nonharsh mothers did (Joosen, Mesman, Bakermans-Kranenburg, & Van IJzendoorn, 2013b).

Aberrant basal activity and/or stress reactivity may be a sign of ANS malfunction. Ideally, the ANS helps individuals adapt to changing circumstances and maintain stability through change, a process called allostasis (McEwen & Seeman, 1999). But when stressful environmental demands are chronic or too frequent, physiological responses may become dysregulated and have detrimental consequences, a condition referred to as allostatic load (Beckie, 2012; Sterling & Eyer, 1988). Maltreating parents may be especially susceptible to allostatic load, given that they tend to live in more stressful circumstances than nonmaltreating parents, for example, having more often been maltreated as children themselves (Pears & Capaldi, 2001), having a lower income (Whipple & Webster-Stratton, 1991), more often being unemployed, and experiencing more personal stress (Stith et al., 2009). Allostatic load may limit parents’ abilities to respond to and downregulate stress (McEwen, 1998), including stressful signals from their children.

Anomalous ANS functioning as a correlate of child maltreatment was first examined in the 1970s (Disbrow, Doerr, & Caulfield, 1977). In their seminal study, Frodi and Lamb (1980) measured HR, blood pressure, and SC in abusive and nonabusive mothers as they viewed videos of a crying/smiling infant. The Frodi and Lamb findings sparked additional interest in the role of autonomic stress reactivity and (risk for) child maltreatment, leading to other studies on this topic (e.g., Casanova, Domanic, McCanne, & Milner, 1992; Crowe & Zeskind, 1992; Friedrich, Tyler, & Clark, 1985; Pruitt & Erickson, 1985; Stasiewicz & Lisman, 1989; Wolfe, Fairbank, Kelly, & Bradlyn, 1983). A narrative review summarizing the results of these early studies concluded that maltreating/at-risk participants exhibited greater autonomic reactivity to stressors compared to their nonmaltreating/low-risk counterparts (McCanne & Hagstrom, 1996). However, findings were notably mixed, possibly because studies varied with respect to sample characteristics (e.g., ranging from maltreating parents to nonparents at risk for child maltreatment), types of standardized stressors used (e.g., ranging from recordings of infant cry sounds to nonchild-related tasks such as solving anagrams), and the autonomic measures assessed (e.g., SC, HR). Moreover, data from recent studies have failed to support the notion that maltreating/at-risk participants exhibit greater autonomic reactivity to stressors (Crouch et al., 2015; Reijman et al., 2014, 2015).

Considering the differences in methodology and findings across studies, and the long lapse since the last review (McCanne & Hagstrom, 1996), the present study was designed to provide an updated narrative review of the literature with a focus on the following research questions: (1) Is (risk for) child maltreatment associated with (a) higher baseline cardiovascular activity, (b) higher baseline sympathetic activity, and/or (c) lower baseline parasympathetic activity? (2) Is (risk for) child maltreatment associated with (a) greater cardiovascular reactivity, (b) greater sympathetic reactivity, and/or (c) blunted parasympathetic reactivity to stressors? The qualitative approach of the narrative review offers a descriptive synthesis of the evidence and allows for an examination of methodological similarities and differences across studies (Petticrew & Roberts, 2006). On the other hand, narrative reviews cannot quantitatively assess the *combined* effect size across studies. A single study may lack statistical power to detect significant differences between groups, and meta-analysis is a tool to assess overall effects across studies. Therefore, we also conducted two sets of meta-analyses in which we distinguished between HR as a mixed index of the ANS (i.e., under both sympathetic and parasympathetic influence), SC as a sympathetic index, and HRV (i.e., respiratory sinus arrhythmia [RSA]; root mean square of successive differences [RMSSDs]) as a parasympathetic index. Based on conclusions from the abovementioned review (McCanne & Hagstrom, 1996), we hypothesized that maltreating and at-risk individuals (compared to nonmaltreating/low-risk individuals) would show (1) higher baseline HR and SC and *lower* baseline HRV and (2) greater HR and SC stress reactivity (defined as an increase in activation relative to baseline) but *smaller* decreases in HRV in response to stressors. Furthermore, we examined variables that might explain differences in effect sizes across studies. Identification of moderators may explain divergent results and provide valuable directions for future research. Specifically, we looked at the following variables as potential moderators: (a) parenting status (whether participants were parents or not), (b) maltreatment status (whether participants had been substantiated for maltreatment or had been identified as at risk), (c) maltreatment type (physical abuse only vs. inclusion of neglect), (d) presentation of stimulus (auditory, visual, or real life), (e) the percentage of women in the sample, (f) sample size, and (g) year of publication. Power analyses were performed to evaluate the adequacy of the sample sizes of individual studies.

## Method

### Literature Search and Inclusion Criteria

We used four search methods in order to retrieve relevant studies. Specifically, we searched the databases Embase, PsycInfo, PubMed, and Web of Science using the following search terms: (“child maltreatment” OR “child abuse” OR “child neglect” OR “physical abuse” OR “physical neglect” OR “emotional abuse” OR “emotional neglect”) AND (parent* OR mother* OR father* OR caregiv* OR risk) AND (autonomic OR physiolog* OR cardiovascular OR HR OR “blood pressure” OR DBP OR SBP OR respirat* OR RR OR HRV OR amylase OR sAA OR sympathetic OR electrodermal OR “skin conductance” OR SCL OR SCR OR parasympathetic OR vagal OR RSA).^1^ Second, these same terms were used to assess potentially eligible dissertations and conference proceedings. Third, we searched Web of Science for references to pioneering articles (i.e., Frodi & Lamb, 1980; McCanne & Hagstrom, 1996). Finally, the reference sections of eligible articles and dissertations were checked for additional potentially eligible papers. Eligibility was based on three main inclusion criteria: (1) The sample consisted of parents with substantiated child maltreatment or participants (parents or nonparents) at high risk for child maltreatment as assessed by a validated instrument (e.g., Child Abuse Potential Inventory [CAP Inventory]) or defined as such by the authors based on a substantial number of risk factors, (2) at least one index of the ANS was measured, (3) the physiological measurement included ANS baseline activity and/or stress reactivity. The stress-invoking stimulus could be child related (e.g., video of a crying infant) or nonchild related (e.g., having to complete a series of anagrams).

From the 1,142 studies obtained through the search of electronic databases, a sample of 150 abstracts was randomly selected in order to establish intercoder reliability with respect to decisions about inclusion in the narrative review and meta-analyses. Two of the authors (R.H. and S.R.) independently coded the 150 abstracts as either *not eligible* or *eligible* (i.e., selected for inclusion). When abstracts were potentially eligible but did not provide sufficient information to determine eligibility, the full text articles were retrieved and coded. The two authors reached 100% consensus on studies coded as eligible. Having established adequate intercoder reliability, the remaining abstracts obtained from the literature search were divided between R.H. and S.R. for independent coding with respect to inclusion versus exclusion. In the case of multiple eligible publications reporting (partly) on the same sample, only the publication with the most available physiological data was included. This ensured that every participant was represented just once in each meta-analysis performed in the present study. For instance, Reijman et al. published two papers on autonomic (re)activity in a largely overlapping sample of maltreating mothers (2014, 2015). The latter included one autonomic measure, namely, salivary alpha amylase, while the former included four more common ones, that is, HR, vagal tone, pre-ejection period (PEP), and SC, and was therefore selected for inclusion in the meta-analyses. Twelve studies published between 1977 and 2015 were identified as eligible for inclusion in the present study: 11 included ANS baseline measures and 11 included ANS stress reactivity (10 studies reported both ANS baseline measures and ANS stress reactivity and thus were included in both meta-analyses). Narrative reviews of these 12 studies are provided below (see also Table 1).

**Table 1.** Summaries of Reviewed Studies.

Study	Sample Size	Parenting Status	Maltreatment Status	Cutoff Scores	Maltreatment Subtype	Autonomic Measures	Stressor	Relevant Findings
Disbrow, Doerr, and Caulfield (1977)	83	Parents	Substantiated	N/A	Abuse and neglect	HR, SC	Videos of stressful dyadic interactions	N/I at rest *ns* for reactivity
Frodi and Lamb (1980)	28	Parents	Substantiated	N/A	Abuse	HR, DBP, SC	Video crying infant	*ns* at rest ↑ HR, SC, ↓ DBP reactivity
Wolfe, Fairbank, Kelly, and Bradlyn (1983)	14^a^	Parents	Substantiated	N/A	Abuse	HR, RR, SC	Videos of parent-child conflict situations	N/I at rest ↑ RR, SC reactivity, *ns* for HR
Friedrich, Tyler, and Clark (1985)	42	Parents	Substantiated	N/A	Abuse and neglect	HR, FBV, SC	Infant cry sound	*ns* at rest *ns* HR, FBV reactivity; ↑ SC sec above baseline
Pruitt and Erickson (1985)	44	Nonparents	High risk (CAPI)	Upper 33% (≥9.1) Lower 33% (≤4.0)	Physical abuse	HR, SC	Video crying infant	N/I at rest ↓ HR reactivity, SC *p* > .05
Stasiewicz and Lisman (1989)	32	Nonparents	High risk (AAPI)	Upper 30% Lower 30%	Abuse	DBP	Infant cry sound and smoke alarm	*ns*
Casanova, Domanic, McCanne, and Milner (1992)	30	Parents	High risk (CAPI)	>166 <66	Physical abuse	HR, SC	Stressful film	*ns*
Crowe and Zeskind (1992)	30	Nonparents	High-risk (CAPI)	Upper 28% (*M* = 283, *SD* = 40.7) Lower 28% (*M* = 53, *SD* = 50.4)	Physical abuse	HR, SC	Infant cry sounds	*ns* at rest ↑ HR reactivity (decrease) ↑ SC reactivity to phonated sounds
Laud (1997)	72	Nonparents	High risk (CAPI)	≥166 ≤63	Physical abuse	HR, DBP, SBP	Infant cry sound	*ns*
Creaven, Skowron, Hughes, Howard, and Loken (2014)	104	Parents	substantiated	N/A	Abuse and neglect	HR, RSA	N/A	↑ HR, ↓ RSA at rest
Reijman et al. (2014)	80^b^	Parents	Substantiated	N/A	Abuse and neglect	HR, RMSSD, PEP, SC	Infant cry sounds	N.s. at rest ↓ SC, ↑ PEP reactivity, HR, RMSSD *p*s > .05
Crouch et al. (2015)	48	Parents	High risk (CAPI)	>166 <166	Physical abuse	HR, RSA	Anagrams	↑ HR, ↓ RSA at rest ↓ HR, RSA reactivity

*Note.* Relevant findings are reported for maltreating or at-risk populations relative to nonmaltreating or low-risk control groups, at *p* < .05. CAPI = Child Abuse Potential Inventory; AAPI = Adult-Adolescent Parenting Inventory; HR = heart rate; SC = skin conductance; RSA = respiratory sinus arrhythmia; FBV = finger blood volume; DBP = diastolic blood pressure; SBP = systolic blood pressure; RMSSD = root mean square of successive differences; PEP= pre-ejection period; RR = respiration rate; *ns* = not significant; N/A = does not apply; N/I = no information reported.

^a^For SC results in Wolfe et al., *N* = 10. ^b^For PEP results in Reijman et al., *N* = 77

When participants were exposed to multiple stressors/stimuli (Casanova et al., 1992; Friedrich et al., 1985; Frodi & Lamb, 1980; Pruitt & Erickson, 1985), we selected one stressor from each study for inclusion in the meta-analyses. This was done for several reasons. First, it ensured that each participant would be represented only once in each meta-analysis. Including multiple effect sizes for samples exposed to multiple stressors would have given more weight to those samples than to others. Alternatively, we could have calculated one combined effect size for the multiple stressors, but this strategy would have made studies less comparable and it would also have made moderator analyses for *presentation of stimulus* impossible. The hierarchy of criteria used to select a single stressor from studies that presented multiple stressors was as follows: (a) psychosocial stimuli such as cry sounds were preferred over physical tasks such as immersing a foot in ice-cold water, (b) child-related stimuli were considered more relevant than nonchild-related stimuli, and (c) stress-invoking stimuli were selected over nonstress-invoking stimuli. These eligibility criteria led to the inclusion of the stressful film task in the Casanova, Domanic, McCanne, and Milner (1992) study, the audiotaped infant crying in the Friedrich, Tyler, and Clark (1985) study, the video of the crying infant in the Frodi and Lamb (1980) and Pruitt and Erickson (1985) studies, and the stressful scenes in the Wolfe, Fairbank, Kelly, and Bradlyn (1983) study.

### Narrative Review

#### Parents with substantiated child maltreatment

Six studies included parents who had been substantiated for abuse and/or neglect of their children. Creaven, Skowron, Hughes, Howard, and Loken (2014) recruited 52 mother–child dyads in which the mother had been a perpetrator of child abuse or neglect. Child protective services (CPS) records were coded using the Maltreatment Classification System (Barnett, Manly, & Cicchetti, 1993). The group was compared to 52 mother–child dyads without previous CPS records. Maltreating and nonmaltreating mothers did not differ on age, employment status, child age, or child sex, but maltreating mothers were less educated and had lower household incomes. For a baseline assessment in the lab, dyads were seated together on a couch under dim lights and watched a low-action animation film for 5 min. HR and RSA were measured in both mother and child. Maltreating mothers showed significantly higher HR and lower RSA at baseline than nonmaltreating mothers. Although dyads’ HR and RSA were measured during a joint task, the study did not assess autonomic *stress* reactivity.

The remaining five studies included measurements of autonomic responses to stressful child-related stimuli. Disbrow, Doerr, and Caulfield (1977) recruited 22 physically abusive and 24 neglectful parents via CPS. Of the total sample, 63% were mothers. Maltreating parents were matched to a nonmaltreating comparison group on age, education, ethnicity, relationship status (single vs. couple), and children’s age. The comparison group was screened to verify they had not been previously reported to CPS. In the lab, parents watched a videotape of interactions between a mother, father, and child of the same race as themselves. The tape included pleasant and stressful interactions. For the baseline assessment, neutral colors were presented before the start of the tape as well as in between interaction scenes. No information was reported on whether groups differed in autonomic arousal during baseline. Information on differential reactivity from baseline to the stressful interaction scenes was reported only for HR. The change in HR from baseline to the stressful interaction scenes did not differ significantly for maltreating and comparison parents.

Frodi and Lamb (1980) included 14 physically abusive and 14 comparison mothers. Abusive mothers were recruited through Parents Anonymous and all admitted to having abused at least one of their children. The comparison group was individually matched to the abuse group on age, marital status, social class, number of children, and children’s age. Participants watched two videotapes with three 2-min segments each (also used by Pruitt & Erickson, 1985, see below). The first and last segment of each tape showed an infant quiescent but alert. The middle segment of one tape showed the same infant smiling and cooing, while the middle segment of the other tape showed the infant crying. The order of presentation of the two tapes was counterbalanced. HR, SC, and diastolic blood pressure (DBP) were measured during a 2-min rest period and the first and last 30 seconds of each video segment. For reactivity analyses, the last 30 seconds of the first segment showing the infant quiescent was used as a baseline from which change scores were calculated. Abusive and nonabusive mothers did not differ significantly on baseline levels for any of the autonomic measures. In response to the crying infant, abusive mothers showed greater HR and SC increases, but smaller DBP increases than nonabusive mothers.

Wolfe et al. (1983) used videotaped interactions of stressful mother–child interactions as a stressor. Participants included seven maltreating mothers (who had been referred to a treatment program by the local child welfare agency after verification of child abuse) and seven comparison mothers. The groups were individually matched on education, income, number of children, children’s age, and parent-reported child behavior problems. After a 5-min resting baseline, mothers watched a 3-min videotape with 12 scenes of mother–child interactions. Some interaction scenes were stressful (e.g., dyadic conflicts), whereas others were not (e.g., mother and child playing together). After that, a 5-min post-task baseline was recorded. HR, respiration rate (RR), and SC responses were measured during the pre- and post-task baseline and while viewing the interaction scenes. Four scenes were rated as stressful by more than 65% of mothers. Although means and standard deviations (*SD*s) for autonomic values at baseline and during the stressful scenes were displayed for both abusive and nonabusive mothers, whether the groups differed significantly on autonomic arousal at baseline was not reported. Using baseline levels as a covariate, abusive mothers showed higher SC and RR during the stressful scenes than nonabusive mothers. There were no effects for HR.

Friedrich et al. (1985) and Reijman et al. (2014) used infant cry sounds as a stressor. Friedrich et al. (1985) had a sample of abusive (*n* = 14), neglectful (*n* = 13), and comparison mothers (*n* = 15). Maltreating mothers had been substantiated for abuse or neglect within the past year. The comparison group received financial aid from the county welfare office, and during the time they were receiving the assistance, no reports of abuse or neglect were filed against them. The three groups did not differ on age, education, income, marital status, or children’s age, although abusive and neglectful mothers on average had more children than comparison mothers. Mothers listened to a 9-min audiotape on which 1-min sounds of white noise, a tone, and infant crying were alternated. Results for the cry sound were selected for this review and the meta-analyses (see inclusion criteria described above). The order of presentation of the segments was counterbalanced, but the cry sound was always preceded by the nonstressful white noise. HR, finger blood volume (FBV), and SC were measured during a 7-min baseline and throughout the presentation of the audiotape. There were no significant differences between the groups on any of the measures at baseline. For HR and FBV, reactivity to the cry sound was analyzed as the difference between mean values at baseline and during the cry. There were no significant differences among the maltreatment groups for HR reactivity or FBV reactivity. For SC, reactivity was analyzed in two ways: as the increase from the last 10 seconds of white noise to the first 10 seconds of the cry (deflections) and as the total number of seconds SC was higher during the cry than during baseline. There were no significant differences between groups in their SC deflections, but there was a difference between groups on the number of seconds above baseline. Particularly during the second cry segment, both the abusive and neglectful groups (compared to the comparison group) showed more sustained SC increases relative to baseline.

Reijman et al. (2014) recruited a sample of maltreating mothers through a mental health clinic, where mothers received therapy focusing on their parenting problems. Incidents of abuse and neglect were coded from CPS records. All mothers were found to be neglectful, while about half were also physically abusive. Nonmaltreating mothers were recruited from a different subdivision of the same mental health clinic, where their children were in therapy for a developmental or learning disorder. In this group, the Maternal Maltreatment Classification Interview (Cicchetti, Toth, & Manly, 2003) was conducted to verify the absence of maltreatment incidents. Physiological data were available for 42 maltreating and 38 nonmaltreating mothers. The groups did not differ on ethnicity, education, medication intake, number of children, or whether children were clinically diagnosed, but maltreating mothers and their children were significantly younger, more maltreating mothers smoked, and fewer exercised as compared to the nonmaltreating group. These variables (age, smoking, and exercise habits) were controlled for in the analyses. After watching neutral images during a 5-min baseline assessment, they listened to nine 10-s infant cries of varying pitches. HR, PEP, HRV (RMSSD), and SC were measured throughout. No significant differences were found between the groups for any of the autonomic variables at baseline. From baseline to the cry sounds, maltreating and nonmaltreating mothers showed similar HR and RMSSD responses, but there was an effect of maltreatment status on PEP reactivity, with maltreating mothers showing a nonsignificant PEP decrease, while the comparison group showed a nonsignificant PEP increase. Finally, maltreating mothers showed less SC reactivity than nonmaltreating mothers.

##### Summary

Of these six studies with maltreating parents, one provided evidence supporting the association between child maltreatment and higher resting HR and lower parasympathetic activation (Creaven et al., 2014). Information on autonomic activity at baseline was not reported by Disbrow et al. (1977) or Wolfe et al. (1983). The three remaining studies found no significant associations between child maltreatment status and autonomic baseline levels (Friedrich et al., 1985; Frodi & Lamb, 1980; Reijman et al., 2014).

Regarding the association between child maltreatment status and reactivity to stressful stimuli, evidence was mixed as well. In the Frodi and Lamb (1980) and Wolfe et al. (1983) studies, effects for two of the three autonomic measures supported the link between child maltreatment and increased sympathetic stress reactivity. Friedrich et al. (1985) found that abusive and neglectful mothers (relative to comparison mothers) showed more sustained increases in SC during a cry sound as compared to baseline, but there were no significant differences between groups in SC deflections, HR reactivity, or FBV reactivity. In Reijman et al. (2014), only the differential direction of PEP responses to infant crying suggested slightly more sympathetic reactivity in maltreating mothers. However, maltreating mothers showed weaker SC responses than nonmaltreating mothers, indicating less sympathetic reactivity, while there were no significant effects for HR or RMSSD. Finally, autonomic stress reactivity did not distinguish abusive from comparison parents in Disbrow et al. (1977).

#### Parents and nonparents at risk for child abuse

Six studies assessed the risk for committing child abuse in parents and nonparents. Five of the six studies used a validated instrument designed to assess risk for child physical abuse, namely, the CAP Inventory (Milner, 1986; Milner & Wimberley, 1979). The CAP Inventory is a self-report questionnaire that consists of 160 statements to which respondents are asked to indicate whether they agree or disagree. It consists of an abuse potential scale (77 items), six factor scales (e.g., distress, rigidity, unhappiness, various interpersonal problems), and three validity scales to detect if respondents answered randomly, faked good (i.e., denied problems), or faked bad (i.e., exaggerated problems). Adequate psychometric properties, including construct validity, internal consistency, and stability over time, have been demonstrated across numerous samples (see Milner, 2004, for a review, but see Voorthuis et al., 2014).

Casanova et al. (1992) recruited 151 parents from day-care and social service agencies. All were screened with the CAP Inventory. Respondents with valid answers were included in the high-risk group if they scored 166 or higher (the signal detection cutoff score), while those who scored below the median norm abuse score of 66 were considered low risk. Fifteen high-risk mothers were individually matched with 15 low-risk mothers on ethnicity, age, marital status, number of children, and children’s age. The two groups of mothers were exposed to a series of nonchild-related stimuli, namely, a cold pressor task, a stressful film, unsolvable anagrams, and car horn sounds. For each task, HR and SC were measured the minute prior to stimulus onset (baseline), the minute of stimulus presentation, and the minute after stimulus completion. Results for the stressful film were selected for inclusion in this review and the meta-analyses (see inclusion criteria described above). The stressful 1-min film displayed two industrial accidents. There were no significant differences in HR during baseline, while no information was reported on significant differences between groups for SC baseline levels. The stressful film evoked a stress response on both ANS measures, but no significant differences between high- and low-risk mothers in HR reactivity and SC reactivity (from baseline to film exposure) were found.

Crouch et al. (2015) studied a sample of 48 parents, of which 28 were women. Parents with valid response patterns on the CAP Inventory were classified as high risk if their CAP abuse score was at or above the signal detection cutoff score of 166, while those with a score below 166 were considered low risk. The two groups did not differ significantly on age, gender, education, annual household income, marital status, or number of children. More high-risk parents were African American; however, race/ethnicity was not associated with any of the outcome measures. All parents completed a computer task which required them to solve as many anagrams as possible in 3 min. Participants were randomly assigned to either a difficult anagram condition or an easy anagram condition. HR and RSA were measured during a 3-min baseline and during the anagram task. At baseline, high-risk parents showed higher HR and lower RSA than low-risk parents. In response to the anagram task, HR and RSA of high-risk parents did not change, while low-risk parents showed an increase in HR and a decrease in RSA. Difficulty of the anagram task did not moderate patterns of change in HR or RSA over time.

The four remaining studies sampled nonparents and used child-related stimuli. Pruitt and Erickson (1985) recruited 61 nonparents who were 30 years of age or younger. Based on the CAP Inventory, placement in the high- versus low-risk groups was determined by taking the upper and lower 33% of nonweighted abuse scores. Twenty-two participants (14 women) were classified as high risk (nonweighted abuse score > 9.1) and 22 participants (16 women) were classified as low risk (nonweighted abuse score ≤ 4). No matching of the groups on demographics was reported. Participants were shown two videotapes of 6 min each. One video showed a 5-month-old female infant first quiescent but alert (2 min), smiling and cooing (2 min), and then again quiescent (2 min), while the other video showed the same infant quiescent (2 min), crying (2 min), and quiescent (2 min). The same videotapes had been used by Frodi and Lamb (1980; see above). Whether the video with the smiling or the crying infant was shown first was counterbalanced within women/men in the low-/high-risk groups. HR and SC responses were measured 2 min before and throughout the videotapes. Results were reported in peak HR and peak SC rather than mean levels. Patterns of autonomic functioning across the smiling/crying videotapes were analyzed; however, risk group differences in baseline autonomic activity and reactivity specific to the crying infant were not reported. Compared to low-risk participants, high-risk participants exhibited significantly higher peak HR across the video segments. Moreover, high-risk participants exhibited lower HRV across the quiescent, smiling, and crying video segments. There were no significant differences between the low-risk and high-risk groups with respect to SC variability across the quiescent, smiling, and crying videotape segments.

Crowe and Zeskind (1992) screened 284 introductory psychology students for child physical abuse risk using the CAP Inventory. After excluding students whose responses on the CAP Inventory were invalid or incomplete, 30 participants were selected with either high CAP scores (upper 28th percentile of scores; *M* = 283, *SD* = 40.7) or low CAP scores (lower 28th percentile of scores; *M* = 53, *SD* = 50.4). Both groups consisted of eight men and seven women and did not differ on age, ethnicity, income, or reported history of abuse. Participants listened to two audio recordings, one with four 10-s phonated infant cry sounds and one with four 10-s hyperphonated infant cry sounds. The first tape, containing either phonated or hyperphonated cries, was repeated twice. After a 10-min rest, the remaining tape of phonated/hyperphonated infant cry sounds was played twice. Order of presentation of the phonated/hyperphonated cries was counterbalanced within men/women in the low-/high-CAP groups. HR and SC were assessed 2 min before stimulus onset and throughout the presentation of the cry sounds. No significant differences between the high- and low-CAP groups were reported for baseline HR or baseline SC. In response to the cry sounds, the high-CAP group showed marginally greater HR changes than the low-CAP group, but in a negative direction, so that the HR of those at risk for child abuse tended to decrease, while that of the low-CAP group did not. The authors also reported a marginally significant interaction effect of CAP risk status and cry type (phonated vs. hyperphonated) on SC responses, such that the high-CAP group showed somewhat higher SC responses to the phonated sounds than the low-CAP group. There were no risk group differences in SC reactivity to the hyperphonated cry sounds.

Laud (1997) also used the infant cry sound as a stress-evoking stimulus. Participants were randomly chosen from a larger pool (*N* = 199) of unmarried, nonparent, female psychology students who were screened for health (including cardiovascular) and hearing concerns. Based on CAP Inventory abuse scores, 38 respondents were classified as high risk (CAP abuse score ≥ 166) and 34 respondents were classified as low risk (CAP abuse scores ≤ 63). The high-risk and low-risk groups did not differ on ethnicity, age, or education. After a 4-min resting baseline, participants listened to an infant cry sound that lasted 8 min. HR was recorded throughout the baseline and the cry sound presentations, and DBP and systolic blood pressure (SBP) were measured every 2 min. CAP risk groups did not differ on any of the baseline autonomic measures or in their autonomic response from baseline to the cry sounds.

Stasiewicz and Lisman (1989) used the Adult-Adolescent Parenting Inventory (AAPI; Bavolek, Kline, McLaughlin, & Publicover, 1979) to assess child abuse risk in a sample of male, unmarried, nonparent undergraduate students. Participants who obtained scores in the upper 30% of the AAPI distribution of scores were classified as high risk (*n* = 16) and those with scores in the lower 38% of the distribution of AAPI scores were classified as low risk (*n* = 16). No information on whether the risk groups were demographically matched was reported. After a 6-min resting baseline, participants were either exposed to an audio recording of the cry sounds of a medically at-risk infant or the sound of a smoke alarm. Results were reported for the two stressors combined, so that examination of data specific to the infant cry sounds was not possible. The volumes required to evoke similar levels of aversiveness in response to the infant cry sound and the smoke alarm sound were determined in a pilot study. Infant cries and the smoke alarm sounds were presented for 3 min each and were repeated 3 times with 2-min breaks between presentations. DBP was assessed as an index of ANS activation. High-risk and low-risk participants did not differ significantly with respect to baseline DBP or in their DBP response to the infant cry/smoke alarm sounds.

##### Summary

Results of significance testing in most of the studies with at-risk samples found no significant evidence for a link between risk for child abuse and autonomic activity at baseline (Casanova et al., 1992; Crowe & Zeskind, 1992; Laud, 1997; Stasiewicz & Lisman, 1989), or autonomic reactivity to stressful child- or nonchild-related stimuli (Casanova et al., 1992; Laud, 1997; Stasiewicz & Lisman, 1989). Information on autonomic baseline differences was partially (or not explicitly) reported in Casanova et al. (1992) and Pruitt and Erickson (1985). Consistent with our hypotheses, Crouch et al. (2015) found that high-risk parents showed higher HR and lower RSA at baseline. Also, as expected, the high-risk group did not exhibit a decrease in RSA in response to a stressful task, whereas the low-risk group did. However, in contrast with our hypotheses, high-risk parents showed less HR reactivity to the stressful task than low-risk parents. Crowe and Zeskind (1992) found greater HR reactivity to cry sounds in the high-risk group, but the reactivity constituted a decrease rather than an increase in arousal. In Pruitt and Erickson (1985), high-risk participants showed no HR change in response to a video of a crying infant, while the low-risk group showed an HR decline. There were no other risk group differences in autonomic reactivity to the stressors used in the reviewed studies.

##### Conclusion

Across both sets of studies on parents with substantiated maltreatment and individuals at risk for abuse, only two studies provided evidence (based on significance testing) supporting the notion that maltreating/at-risk individuals experience heightened HR and lower RSA activation at baseline (Creaven et al., 2014; Crouch et al., 2015, respectively). These two studies varied in their sample characteristics (substantiated maltreatment vs. at-risk status), maltreatment type (abuse and neglect vs. risk for physical abuse), gender ratio (mothers only vs. mothers and fathers), sample size (*N* = 104 vs. *N* = 48), and baseline procedure (watching a video in the presence of their child vs. resting in solitude). Both Creaven et al. and Crouch et al. measured HR and RSA, but studies that did *not* find significant group differences on ANS baseline activity also included HR and HRV as outcome measures (e.g., Reijman et al., 2014). Synthesis of the findings is further complicated by the fact that several studies did not report statistical tests examining whether the maltreating/at-risk groups differed from their comparison groups on ANS baseline values (although in some cases, descriptive statistics for baseline values were presented and effect sizes could be calculated and included in a meta-analysis; see below).

Regarding ANS stress reactivity as a risk factor for child maltreatment, the least equivocal findings were presented by Frodi and Lamb (1980) and Wolfe et al. (1983). Both samples consisted of physically abusive parents, all mothers, who were presented with stress-invoking, child-related videotapes. Common autonomic measures were HR and SC, and abusive mothers showed heightened SC stress reactivity in both studies. Frodi and Lamb (1980) additionally found greater HR reactivity in the abusive group while Wolfe et al. (1983) did not. The remaining three studies with maltreating samples (which included neglectful parents) and none of the studies with at-risk samples reported differential sympathetic reactivity based on significance testing. This tentatively suggests that increased sympathetic reactivity is a risk factor specific to substantiated physical abuse.

In the set of studies using the CAP Inventory, Crouch et al. (2015) found blunted RSA reactivity in the high-risk group, in line with our hypothesis. Operational variation was also present in the “at-risk” studies, with cutoff scores being criterion referenced (i.e., signal detection score of 166) in some studies and norm referenced (e.g., upper vs. lower 33 percentile of sampled scores) in others. Such differences in methodology may help explain the variability of findings observed across studies.

Moreover, small sample sizes may have contributed to instability of results across the studies reviewed. For example, use of small samples may produce exaggerated effects, low positive predictive power, and increased risk of either Type I or Type II errors (for a discussion of these issues, see Button et al., 2013). This makes it difficult to draw conclusions from individual studies based on significance testing. A systematic review of effect sizes across studies is needed to better understand this literature.

### Meta-Analytic Procedures

Although the narrative review conveys the similarities and differences of the methods and the results of significance testing across studies, it does not quantitatively analyze the strength of the effects observed. Results of the narrative review revealed seemingly contradictory findings (based on significance testing) among as well as within studies (e.g., Frodi & Lamb, 1980; Reijman et al., 2014), an observation that is consistent with that of an earlier review (McCanne & Hagstrom, 1996). Meta-analysis is thus warranted in order to estimate the overall effects for the relations between (risk for) child maltreatment and autonomic baseline activity as well as stress reactivity and to test whether effects may be moderated by sample or study characteristics.

### Moderators

For the meta-analyses, we coded two types of moderators: sample related and procedure related. Sample-related moderators were *maltreatment status* (categorical: substantiated maltreatment vs. risk for physical abuse) and *percentage of women* in the sample (continuous). Procedural characteristics were *presentation of stressor* (categorical: auditory vs. visual vs. real-life stimuli), *publication year* (continuous), and *sample size* (continuous). Two potential moderators, *parenting status* (whether participants were parents or not) and *maltreatment type* (studies that focused on [risk for] physical abuse vs. studies that included neglect), were excluded because of their high overlap (83% in both cases) with *maltreatment status* in the current set of studies. Interrater reliability of the coding of moderators was good, with intraclass correlations for continuous moderators ranging from 0.96 to 1, and κs for categorical moderators ranging from 0.85 to 1.

### Statistical Analyses

We performed meta-analyses on two overall outcomes: the association between (risk for) child maltreatment and ANS baseline activity and the association between (risk for) child maltreatment and ANS stress reactivity. Almost all studies had more than one ANS outcome measure. Therefore, within each of these two sets of studies, we conducted several meta-analyses: one on HR as an index of mixed ANS (re)activity (i.e., under the influence of both the sympathetic and the parasympathetic branch), because it was included in nearly every study; another on SC as a measure of sympathetic (re)activity; and one for indices of HRV (i.e., RSA and RMSSD) which reflect parasympathetic activity and correlate highly (Goedhart, Van der Sluis, Houtveen, Willemsen, & De Geus, 2007). Otherwise, different indices of the same subsystem (e.g., SC and PEP as measures of the SNS) were not combined because their frequent lack of correlation does not confirm the assumption of a single underlying construct (e.g., Reijman et al., 2014; Robinson & Demaree, 2007). As a consequence, the study by Stasiewicz and Lisman (1989) was excluded from the meta-analyses because DBP was its only autonomic measure.

**Figure 1.**
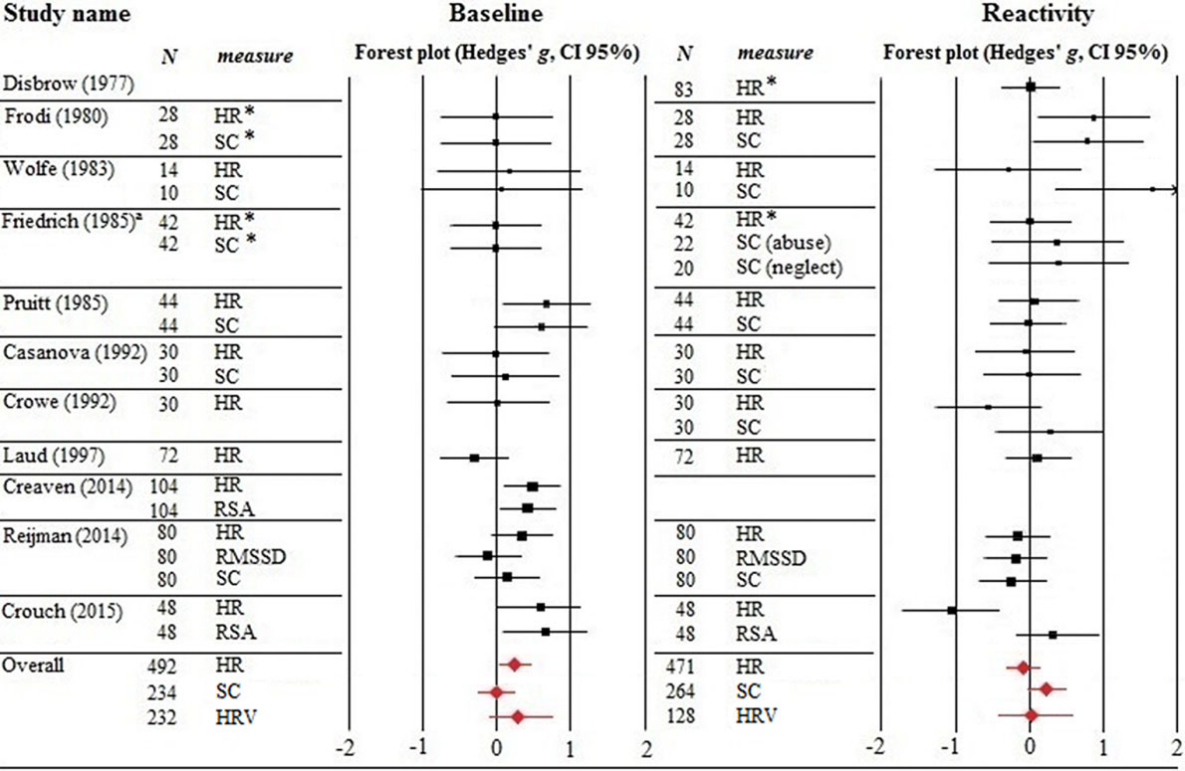
Effect sizes for HR, SC, HRV baseline activity, and stress reactivity of the individual studies. ^a^The sample of Friedrich et al. (1985) consisted of abusive, neglectful, and control mothers. For skin conductance, results for the abusive and neglectful groups were reported separately, so we divided the control group’s *n* by two in order to avoid double representation of participants. HR = heart rate; SC = skin conductance; RSA = respiratory sinus arrhythmia; RMSSD = root mean square of successive differences (measure of vagal tone). Asterisks indicate effect sizes based on *p* = .50 due to lack of statistical details.

Study outcomes were entered in Comprehensive Meta-Analysis (CMA; Borenstein, Rothstein, & Cohen, 2005). When mean values were reported without *SD*s, in most cases, we estimated the *SD*s based on the values for the corresponding autonomic measure in Reijman et al. (2014). Similarly, the *SD*s for the SC means reported in Pruitt and Erickson (1985) were estimated based on Casanova (1990, which contains the *SD*s for Casanova et al., 1992) because SC was reported in micromhos × 10^6^ in both papers. CMA transformed the outcomes into Hedges’ *g* effect sizes, which is appropriate for smaller sample sizes (Cumming, 2012; Lakens, 2013). Effects consistent with our hypotheses were marked positive, while effects in the opposite direction were identified as negative. Reactivity was defined as *increases* (relative to baseline) in HR and SC and decreases in HRV. In the case of findings indicated as nonsignificant but without further statistical details, we assigned a zero effect size at *p* = .50 (Mullen, 1989). These cases are marked with an asterisk in Figure 1. Confidence intervals (CIs) of 95% around the point estimate of every effect size are reported.

Statistics for the combined effect sizes (with 95% CIs) and moderator analyses were drawn from random effect models. Random effect models are based on the assumption that studies differ in their characteristics, and since meta-analytical results are calculated from this assumption, they may be generalized to studies not sampled in the meta-analysis, but belonging to the same population (Hedges & Vevea, 1998). We tested the homogeneity of different sets of effect sizes and moderating effects of categorical variables with the *Q* statistic (Borenstein et al., 2005). Contrast analyses for categorical moderators were conducted only when there were at least two groups with *k* ≥ 4 (Bakermans-Kranenburg, Van IJzendoorn, & Juffer, 2003). Continuous moderators were tested in univariate as well as multivariate regression models, since *year of publication* and *sample size* were correlated (*r* = .56, *p* = .04). We also performed a series of cumulative meta-analyses according to *year of publication*, in which the combined effect size with the addition of each new study was calculated, to further inspect time-related trends.

In the case of significant combined effect sizes, funnel plots were inspected for potential publication bias, that is, the tendency for small studies with nonsignificant or unexpected results to remain unpublished, which would be visually represented by the funnel plot’s asymmetrical base. We calculated a fail-safe number to reflect the number of studies with null results necessary to reduce the effect size to a nonsignificant effect. Finally, we conducted power analyses for individual studies based on the combined effect sizes in the program G-Power 3.1 (Faul, Erdfelder, Lang, & Buchner, 2007), to calculate (1) the sample size required to detect the combined effect size, with α = .05 and a power of .80, and (2) the power of each study to detect the combined effect size, given their sample size and α = .05.

No outliers were found for any of the continuous moderators (standardized *z*-scores <−3.29 or >3.29; Tabachnik & Fidell, 2001). Checks for outliers in effect sizes were done at the level of analysis, that is, for HR, SC, and HRV separately, and revealed no outliers.

## Results

### Child Maltreatment and ANS Baseline Activity

Point estimates and respective CIs of the effect sizes for the outcome measures of each study included in the meta-analysis examining the link between (risk for) child maltreatment and autonomic baseline activity are presented in Figure 1.

For baseline HR, the set of studies (*k* = 10, *N* = 492) was homogeneous, *Q* = 11.81, *p* > .05. The combined effect size was significant (*g* = 0.24, 95% CI [0.03, 0.45], *p* < .05), indicating that perpetration and risk for perpetration of child maltreatment was associated with higher HR levels at baseline. The funnel plot was symmetrical, showing no evidence for publication bias. The fail-safe number was 6, indicating that six null results would be necessary to reduce this meta-analytic finding to a nonsignificant effect. Our power analyses showed that a sample size of *N* = 432 would be required to detect the combined effect size *g* = 0.24. The power of the individual studies to detect this effect size ranged from .11 for the study with the smallest sample size to .33 for the largest sample size. The combined effect sizes for the sets of studies examining the association between (risk for) child maltreatment and SC at baseline (*k* = 6, *N* = 234) and HRV at baseline (*k* = 3, *N* = 232) were not significant (*g* = 0.06 and *g* = 0.30, respectively; see Table 2 for statistical details).

**Table 2.** Combined Effect Sizes for Autonomic Baseline Activity.

	*k*	*N*	*g*	95% CI	*Q^h^*	*Q^c^*
HR	10	492	0.24*	[0.03, 0.45]	11.81	
Maltreatment status						0.13
Substantiated	5	268	0.28	[−0.03, 0.59]	2.49	
At risk	5	224	0.20	[−0.13, 0.52]	8.82	
SC	6	234	−0.003	[−0.27, 0.26]	0.29	
Maltreatment status						
Substantiated	4	160	0.08	[−0.23, 0.39]	0.17	
At risk	2	74	0.02	[−0.43, 0.46]	0.24	
HRV	3	232	0.30	[−0.14, 0.75]	5.50	
Maltreatment status						
Substantiated	2	184	0.17	[−0.38, 0.71]	3.43	
At risk	1	48	0.67	[−0.20, 1.54]		

*Note.* Contrasts were tested for subgroups with *k* ≥ 4. *k* = number of studies; *N* = number of participants; *g* = Hedges’ *g* effect size; CI = confidence interval; *Q^h^ =* homogeneity index; *Q^c^ =* contrast index; HR = heart rate; SC = skin conductance; HRV = heart rate variability.

*p < .05.

There were no moderating effects of *maltreatment status* (*p*s > .05). Due to the low numbers of studies examining baseline HRV, regression analyses for this outcome measure were not conducted. For baseline HR and SC, regression analyses showed no moderating effects for *percentage of female participants, year of publication, or sample size* (*p*s > .05). Cumulative meta-analyses showed no time-related change in effect sizes.

### Child Maltreatment and ANS Stress Reactivity

Point estimates and respective CIs for all outcome measures included in the meta-analysis examining the link between (risk for) perpetration of child maltreatment and autonomic stress reactivity are displayed in Figure 1. The meta-analytical results are summarized in Table 3.

**Table 3.** Combined Effect Sizes for Autonomic Stress Reactivity.

	*k*	*N*	*g*	95% CI	*Q^h^*	*Q^c^*
HR	10	471	−0.10	[−0.36, 0.16]	17.03*	
Maltreatment status						1.16
Substantiated	5	247	0.03	[−0.26, 0.31]	4.83	
At risk	5	224	−0.26	[−0.71, 0.19]	10.84*	
Presentation of stimulus						0.83
Auditory	4	224	−0.09	[−0.35, 0.17]	2.65	
Visual	5	199	0.09	[−0.19, 0.37]	4.13	
Real life	1	48	−1.07	[−1.72, −0.42]		
SC	8	264	0.27	[−0.04, 0.58]	1.21	
Maltreatment status						
Substantiated	5	160	0.48	[−0.14, 1.09]	11.84*	
At risk	3	104	0.07	[−0.31, 0.45]	0.40	
Presentation of stimulus						1.55
Auditory	4	152	0.02	[−0.31, 0.34]	3.03	
Visual	4	112	0.47	[−0.17, 1.11]	7.95*	
Real life	0					
HRV	2	128	0.06	[−0.49, 0.61]	2.30	
Maltreatment status						
Substantiated	1	80	−0.19	[−0.63, 0.25]		
At risk	1	48	0.38	[−0.21, 0.96]		
Presentation of stimulus						
Auditory	1	80	−0.19	[−0.63, 0.25]		
Visual	0					
Real life	1	48	0.38	[−0.21, 0.96]		

*Note.* Contrasts were tested for subgroups with *k* ≥ 4. *k* = number of studies; *N* = number of participants; *g* = Hedges’ *g* effect size; CI = confidence interval; *Q^h^ =* homogeneity index; *Q^c^ =* contrast index; HR = heart rate; SC = skin conductance; HRV = heart rate variability.

*p < .05.

The combined effect size for the sets of studies examining the association between (risk for) child maltreatment and HR stress reactivity (*k* = 10, *N* = 471) was not significant, *g* = −0.10 (see Table 3). The combined effect size estimating the association between perpetration/risk for perpetration of child maltreatment and SC reactivity to stressors (*k* = 8, *N* = 264) was not significant either, *g* = 0.26, 95% CI [−0.1, 0.60], *p* = .15. A nonsignificant effect was also observed for the association between (risk for) child maltreatment and HRV reactivity (*g* = −0.26 for *k* = 2, *N* = 128; see Table 3).

**Figure 2.**
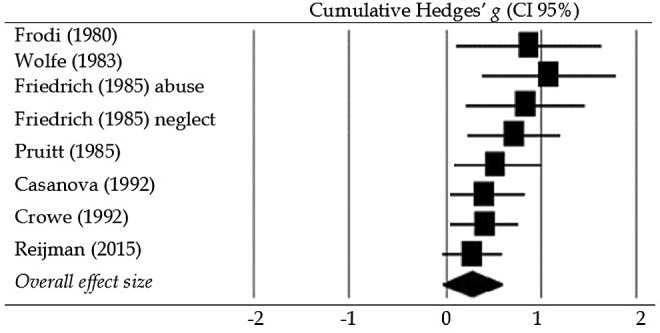
Cumulative effect sizes for skin conductance reactivity. The sample of Friedrich et al. (1985) consisted of abusive, neglectful, and control mothers. For sympathetic reactivity, results for the abusive and neglectful groups were reported separately, so we divided the control group’s *n* by two in order to avoid double representation of participants.

We found no moderating effect of *maltreatment status* or *presentation of stimulus* and there were no significant effect sizes for any of the subgroups (*p*s > .05). No regression analyses for continuous moderators could be conducted for the combined HRV reactivity effect size due to the low number of studies. In univariate models, *year of publication*, *sample size*, and *gender ratio* (% women in the sample) did not moderate the association between (risk for) child maltreatment and HR reactivity (*p*s > .05), while in a multivariate model, *year of publication* and *gender ratio* were significant moderators (*p*s < .01). The regression line for *year of publication* showed a change from positive effect sizes to negative effect sizes over the years. This seems mainly due to an early study that found a large positive effect (Frodi & Lamb, 1980) and a recent study that yielded a strong negative effect (Crouch et al., 2015). The regression line for *gender ratio* showed that samples with lower percentages of women were associated with more negative effect sizes. For SC reactivity, *year of publication* and *sample size* predicted effect sizes in univariate models (*p*s < .03), with effect sizes decreasing as publication year and sample size increased. However, in a multivariate model, neither *year of publication*, *sample size*, nor *gender ratio* was significant (*p*s > .46). Cumulative meta-analyses showed that for SC reactivity, with each aggregated study after Frodi and Lamb (1980) and Wolfe et al. (1983), the combined effect size further approached a null effect, which is displayed in Figure 2.

## Discussion

Results from our meta-analyses are consistent with the notion that maltreating parents and adults at risk for child maltreatment perpetration (relative to their respective comparison groups) exhibit higher levels of HR activity (*g* = 0.24). This finding supports our first hypothesis and converges with conclusions drawn from an earlier review (McCanne & Hagstrom, 1996). Higher HR at baseline may be a sign of chronic cardiovascular arousal in maltreating and at-risk participants and suggests an inability to downregulate stress, even in the absence of immediate stressors. Although the precise mechanism remains speculative, the notion that an inability to regulate stress effectively would impair parenting behavior is not hard to conceive.

Maltreating and at-risk adults may show sustained cardiovascular arousal as a result of allostatic load, due to living in an environment that is (perceived as) continuously overly demanding or challenging. This is consistent with literature showing that maltreating parents tend to experience more early and current adversities, such as having experienced childhood maltreatment, unemployment, single parenthood, and low social support (Euser et al., 2013; Stith et al., 2009). Indeed, life adversities are associated with a variety of physiological health risk factors including high resting cardiovascular activity (Friedman, Karlamangla, Gruenewald, Koretz, & Seeman, 2015). We found no significant effects of (risk for) child maltreatment perpetration on pure measures of sympathetic (i.e., SC) or parasympathetic (e.g., RSA) baseline activity, but only a few studies have included pertinent indices (*k* = 6 and *k* = 3, respectively).

Contrary to our expectations, results from our meta-analyses did not support the notion that increased autonomic stress reactivity is associated with maltreatment or risk for maltreatment. As revealed by our narrative review, only two early studies (Frodi & Lamb, 1980; Wolfe et al., 1983) found that abusive mothers exhibited significantly greater sympathetic increases in response to a stressor. Moreover, results from our cumulative meta-analyses revealed that each of the studies examining sympathetic stress reactivity subsequent to Frodi and Lamb (1980) and Wolfe et al. (1983) has attenuated the size of the aggregate effect (see Figure 2). This suggests that the initial studies examining the association between (risk for) child maltreatment and SNS reactivity may have been subject to the *winner’s curse* (Button et al., 2013; Molendijk et al., 2012). That is, it may be that the large effects found in early studies were the result of using small samples that produced inflated estimates of the association between stress reactivity and maltreatment status. Consistent with a *winner’s curse* interpretation, results from larger studies subsequent to Frodi and Lamb and Wolfe et al. have attenuated the aggregate effect to the point that it is no longer significant.

Thus, findings from our stress reactivity meta-analyses diverge from the observations reported in the narrative review by McCanne and Hagstrom (1996). However, this state of affairs is not surprising, given that McCanne and Hagstrom’s review was limited to the earliest studies on this topic, and some of these initial studies (i.e., Frodi & Lamb, 1980; Wolfe et al., 1983) produced the strongest stress reactivity findings. As described above, subsequent studies have failed to replicate these early effects, despite the use of larger samples. Results of our meta-analyses reflect this trend.

Moreover, in their definition of autonomic reactivity, McCanne and Hagstrom (1996) included both increased and prolonged autonomic activation during any circumstance, including resting/relaxation (i.e., baseline) and the presentation of stressors. In contrast, we examined autonomic activity at baseline separately and defined autonomic reactivity as the change in ANS activity from baseline to stress. We chose to focus on these two outcomes because they were most commonly assessed, but some valuable results not represented in our meta-analyses bear mentioning. For instance, parents at risk for child abuse showed a renewed increase in systolic blood pressure after several minutes of listening to a persistent infant cry sound, which could reflect sensitization, whereas low-risk parents did not show signs of sensitization (Laud, 1997). Furthermore, several studies found that maltreating parents showed similar autonomic responses to child signals regardless of whether the signals were positive or negative, whereas nonmaltreating parents exhibited different patterns of autonomic responses depending on the valence of the stimuli (Disbrow et al., 1977; Frodi & Lamb, 1980). Finally, perpetration and risk for perpetration of child maltreatment were associated with persistently higher HR in Disbrow et al. (1977) and Crouch et al. (2015), and Pruitt and Erickson (1985) reported persistently higher peak HR over time regardless of the presence/absence of stressors. Chronically elevated HR in maltreating parents or at-risk participants suggests there may be a ceiling effect, that is, high levels of HR activity beyond which they show no further increases in response to stress (Crouch et al., 2015).

We found no evidence for moderating effects of the categorical variables in either meta-analysis, such as whether maltreatment was substantiated or risk for physical abuse was assessed with the CAP Inventory. Combined effect sizes were predominantly homogeneous, suggesting that effects were similar regardless of maltreatment status and stimulus presentation, but the small cell sizes preclude any firm conclusions. Multivariate regressions showed that year of publication as well as the percentage of women in the sample predicted the effect size for HR reactivity. Later publications and samples with lower percentages of women were associated with more negative effect sizes. Again, these findings should be interpreted with caution as large effects may tilt the regression line disproportionately, given the small number of included studies. Moreover, the moderating effect of several potentially relevant variables could not be tested, either because the cell size for one of the categories was small even after dichotomization (*k* < 4; e.g., socioeconomic status, whether the stressor was child related or nonchild related) or because data were not consistently reported (e.g., ethnicity, participants’ age).

Given the limited number of studies that met criteria for inclusion in our meta-analyses, our findings should be considered tentative. Homogeneity tests and moderator analyses of small sets of studies might easily lead to Type I and Type II errors. Therefore, the results of our homogeneity tests and moderator analyses should be considered with caution. A second limitation is that the meta-analyses examining baseline physiological activity and physiological stress reactivity were done on largely the same set of studies, so our findings for ANS baseline activity and ANS stress reactivity are not independent.

Another important shortcoming is that groups in quasi-experimental designs are often not equivalent from the outset. When we want to ascribe observed differences in autonomic (re)activity to whether participants are maltreating/at-risk or not, insufficient comparability of groups on potential confounding variables is a threat to internal validity (Shadish, Cook, & Campbell, 2002). Not all of the reviewed studies matched their groups on variables such as socioeconomic status or educational level. When groups differed on a potential confounding variable, this was not always controlled for in analyses.

In addition, few of the studies controlled for maltreatment experienced by participants in their own youth, a factor that is related to child maltreatment perpetration (e.g., Pears & Capaldi, 2001) as well as autonomic responsiveness (Casanova, Domanic, McCanne, & Milner, 1994; Heim et al., 2000). Alternative explanations for observed correlations are thus not ruled out. Furthermore, all of the studies included in our meta-analyses used a case-control design, precluding causal inferences about the association between autonomic (re)activity and child maltreatment. Finally, it is important to realize that most studies measured autonomic (re)activity in controlled laboratory settings using standardized stimuli. The generalizability of these findings to ANS responses in naturalistic parenting environments needs to be corroborated.

Despite these limitations, the current article, in our view, makes several important contributions to the field. First of all, reviews may serve the purpose of correcting misconceptions. Our review and meta-analyses raise doubts about the notion that autonomic hyperreactivity to stress is a risk factor for child maltreatment. The early studies on this topic (e.g., Frodi & Lamb, 1980) had a considerable impact, and their findings played an important role in propelling this notion. Our meta-analysis will hopefully assist future researchers in interpreting these early findings within the context of this growing body of research.

Second, the previous and only review on this topic was published in 1996, and the authors of this earlier review, in examining the evidence for the “hyperreactivity hypothesis,” did not distinguish between ANS activity at baseline and reactivity in response to stress, and they grouped distinct autonomic outcomes under the generic concept of physiological arousal. Ours is the first article to use meta-analytic techniques to evaluate the associations between child maltreatment and various components of autonomic activity. By making distinctions among various physiological components, we sought to clarify findings in this area and encourage others to consider these distinctions in future research.

Third, our article provides a much needed summary of the most important methodological shortcomings in this area of research, including the need to distinguish between abuse and neglect and to include both sympathetic and parasympathetic measures. It is noteworthy that the focus of research on the physiology of maltreating parents/at-risk adults has recently shifted from sympathetic to parasympathetic reactivity. In fact, in the last 20 years, only one study included indicators of sympathetic activity while the studies including measures of parasympathetic activity were all conducted within the past few years. We encourage researchers to consider the relevance of including both sympathetic and parasympathetic indices in future studies to clarify the matters discussed above. Overall, the exploratory nature of our meta-analyses therefore serves a heuristic value with the aim to stimulate new research with more specific hypotheses and corresponding methodology (Van IJzendoorn, Bakermans-Kranenburg, & Alink, 2011).

Although this line of research experienced a 15-year gap in activity after the initial wave of studies, the recent resurgence of studies examining ANS activity in at-risk/maltreating individuals suggests a renewed interest. We hope the studies reviewed here serve as an impetus to the field and that future research will build on and expand their scope. For example, making use of ambulatory assessments of parents’ functioning in their home environment may increase the ecological validity of findings (De Geus & Van Doornen, 1996; Kupper et al., 2005). Recent advances in technology allow for noninvasive assessment of autonomic (re)activity unconfined to laboratory settings. More complex operationalizations of child maltreatment would further help advance research in this area. As mentioned, an expansion of the focus on physical abuse to other types of maltreatment such as emotional abuse and neglect could address relevant questions such as whether different subtypes (or combinations of subtypes) of maltreatment are associated with different autonomic response patterns. Inclusion of degrees of maltreatment severity would allow for a shift from a dichotomous to a more dynamic approach. Finally, randomized experiments using biofeedback or other experimental manipulations of ANS functioning could provide insight into the possible causal role of autonomic activity in perpetration of child maltreatment.

Such additions to the field could further support previous suggestions that maltreating parents may benefit from physiology-based stress regulation (e.g., Casanova et al., 1992; Crouch et al., 2015), but currently the field lacks randomized controlled trials on the effectiveness of such intervention components in maltreating or at-risk populations. A more interactive approach that has been found to be effective is an attachment-based, short-term intervention using video feedback, such as the video-feedback intervention to promote positive parenting (Juffer, Bakermans-Kranenburg, & Van IJzendoorn, 2007). A randomized controlled trial with 67 dyads under surveillance for child maltreatment showed that such an intervention was effective in increasing parental sensitivity (i.e., adequate responding to children’s distress; Moss et al., 2011). Future studies could examine whether the effectiveness of similar intervention programs is enhanced by including elements such as biofeedback to improve maltreating parents’ stress regulation.

## Note

1. Our initial intent was to include studies on the association between (risk for) perpetration of child maltreatment and hypothalamic–pituitary–adrenal axis (re)activity, and relevant terms were part of the literature search, but this did not render a sufficient number of studies to be separately meta-analyzed.
